# Tandem Spinach Array for mRNA Imaging in Living Bacterial Cells

**DOI:** 10.1038/srep17295

**Published:** 2015-11-27

**Authors:** Jichuan Zhang, Jingyi Fei, Benjamin J. Leslie, Kyu Young Han, Thomas E. Kuhlman, Taekjip Ha

**Affiliations:** 1 Department of Physics and Center for the Physics of Living Cells, University of Illinois at Urbana-Champaign, Urbana, IL 61801 USA; 2 Department of Materials Science and Engineering, University of Illinois at Urbana-Champaign, Urbana, IL 61801 USA; 3 Department of Biophysics and Biophysical Chemistry, Johns Hopkins University School of Medicine, Baltimore, MD 21205 USA; 4 Department of Biophysics, Johns Hopkins University, Baltimore, MD 21218 USA; 5 Department of Biomedical Engineering, Johns Hopkins University, Baltimore, MD 21205 USA; 6 Howard Hughes Medical Institute, Baltimore, MD 21205 USA

## Abstract

Live cell RNA imaging using genetically encoded fluorescent labels is an important tool for monitoring RNA activities. A recently reported RNA aptamer-fluorogen system, the Spinach, in which an RNA aptamer binds and induces the fluorescence of a GFP-like 3,5-difluoro-4-hydroxybenzylidene imidazolinone (DFHBI) ligand, can be readily tagged to the RNA of interest. Although the aptamer–fluorogen system is sufficient for imaging highly abundant non-coding RNAs (tRNAs, rRNAs, *etc.*), it performs poorly for mRNA imaging due to low brightness. In addition, whether the aptamer-fluorogen system may perturb the native RNA characteristics has not been systematically characterized at the levels of RNA transcription, translation and degradation. To increase the brightness of these aptamer-fluorogen systems, we constructed and tested tandem arrays containing multiple Spinach aptamers (8–64 aptamer repeats). Such arrays enhanced the brightness of the tagged mRNA molecules by up to ~17 fold in living cells. Strong laser excitation with pulsed illumination further increased the imaging sensitivity of Spinach array-tagged RNAs. Moreover, transcriptional fusion to the Spinach array did not affect mRNA transcription, translation or degradation, indicating that aptamer arrays might be a generalizable labeling method for high-performance and low-perturbation live cell RNA imaging.

RNAs play diverse functions in living cells, including delivering genetic information, catalyzing chemical reactions and regulating gene expression at multiple levels^1^,^2^. Recent genome-wide analysis has suggested that inhomogeneous RNA localization within different cellular compartments might be more prevalent than previously appreciated^3^,^4^, resulting in highly localized spatio-temporal modulations in gene expression levels within those subcellular compartments. Compared to biochemical approaches, such as northern blot, quantitative reverse transcription PCR (qPCR), high-throughput RNA sequencing, *etc*., direct visualization of RNAs by fluorescence imaging allows spatial and temporal RNA tracking and is capable of correlating transcription, localization, translation and degradation of RNAs and simultaneously revealing cell-to-cell heterogeneity^5^,^6^,^7^. Nevertheless, unlike imaging proteins, there are not many tools for imaging RNAs. Fluorescence *in situ* hybridization (FISH) utilizes fluorescent dye-conjugated oligonucleotides (fluorescent probes) complementary to target RNAs to directly label RNA molecules and has been widely applied to accurately quantify the expression level and to localize distribution of mRNAs in biological samples^8^,^9^. Introduction of the fluorescence probes usually requires cell fixation and permeabilization fixed cell^8^. Alternative delivery methods compatible with live cell imaging include microinjection^10^,^11^, electroporation^3^, or transfection using polycationic molecules such as liposomes and dendrimers^12^, and membrane permeabilization via cell-penetrating peptides^13^ and streptolysin O^14^,^15^. However, these methods sometimes lead to problems such as cell damage, inhomogeneous probe delivery and inefficient probe annealing to target RNAs^12^.

The second type of RNA imaging utilizes indirect labeling and employs fluorescent fusion proteins and specific protein-RNA interactions, such as the RNA bacteriophage MS2 coat protein system^16^,^17^,^18^, the PP7 bacteriophage system^19^,^20^, the bacteriophage λ N coat protein system^21^, *etc.*^22^,^23^. In those strategies, RNAs of interest are tagged with a “cognate” RNA sequence, often in a tandem array, recognized and bound by interacting proteins with fluorescent protein fusion. There are several potential limitations associated with this type of methods: (1) usually the overexpressed unbound proteins generate high fluorescent background^24^, unless specific measures are taken to reduce the background^22^,^23^,^25^; (2) the resulting large ribonucleoprotein complex has been reported to affect the RNA endogenous degradation in some cases^26^,^27^, and may potentially affect RNA trafficking and localization^11^; (3) the conditions required for maturation of the fluorescent proteins prohibit the application of these approaches to certain biological systems, such as anaerobic species^28^,^29^,^30^.

More direct ways for RNA labeling in living cells mostly use RNA aptamers that can bind small ligands (or “fluorogens”) and activate their fluorescence^31^,^32^,^33^. Among several aptamer-ligand combinations, “Spinach” and analogous systems (Spinach^34^, Spinach 2^35^, RNA Mango^36^, Broccoli^37^, *etc*.) have shown the greatest potential in biochemical assay and live cell imaging^34^,^35^,^36^,^37^,^38^,^39^,^40^,^41^,^42^,^43^,^44^,^45^. Spinach uses a short RNA aptamer (24-2 and 24-2-min RNA sequences, reported by Paige and coworkers^34^; ~100 nucleotides) that exhibits EGFP-like green fluorescence upon binding of 3,5-difluoro-4-hydroxybenzylidene imidazolinone (DFHBI), a fluorogenic ligand that is structurally similar to the EGFP chromophore and is membrane-permeable and nontoxic. Spinch RNA folds into an RNA G-quadruplex structure providing a binding site for the fluorogen^46^,^47^,^48^, and structural stabilization of the fluorogen bound to the G-quadruplex structure is likely responsible for its strong fluorescence enhancement. Although the Spinach system has been used for imaging highly abundant nontranslated RNAs (tRNAs^34^, rRNAs^34^,^35^,^37^ and trinucleotide repeats^35^) and further applied to detect cellular metabolites and proteins^34^,^35^,^40^,^41^,^44^, there have been very few studies on the utility of Spinach in imaging cellular mRNA^42^, and the fluorescence signal was only barely above cellular autofluorescence level, likely due to low abundance of mRNA and the low brightness of a single fluorogen-bound RNA aptamer. To amplify the signal, making a tandem array of these RNA aptamers, as was used for MS2 and PP7 systems^26^,^27^, could be a general solution to improve the brightness of the aptamer-fluorogen systems. However, it remains to be tested whether this strategy is suitable for RNA imaging because making tandem arrays of these RNA aptamers would significantly increase the RNA length which may potentially affect mRNA metabolism.

Here we use Spinach as our model system and fused multiple repeats of the Spinach aptamer in tandem on a single RNA molecule. We applied the tandem Spinach arrays to cellular mRNA imaging. In both *in vitro* measurement and cellular imaging, the Spinach array brought about as high as 17-fold fluorescence enhancement compared to single Spinach aptamer on RNAs, allowing us to image mRNA distributions inside living cells that could have not been achieved with single aptamer tagging. We also characterized the effects of such tandem arrays on mRNA transcription, translation, localization and degradation, and found that the Spinach array does not alter these RNA characteristics, indicating that making tandem repeats of these aptamers might be a generally applicable strategy for mRNA live cell imaging.

## Results

**Figure 1:**
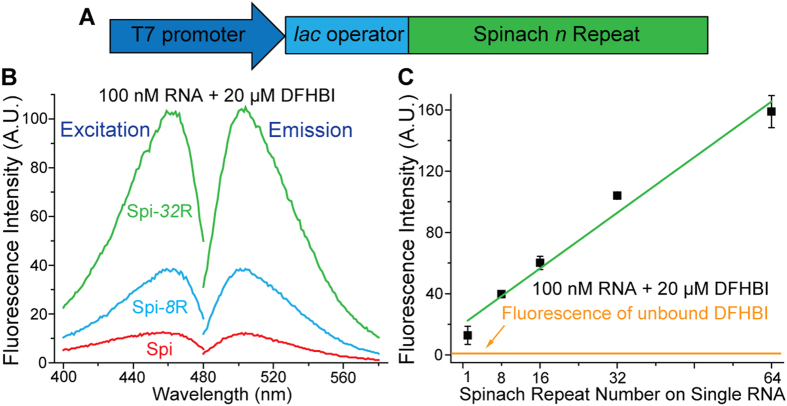
(**A**) Sketch of *in vitro* transcription system for Spinach arrays (Spi-*n*R). (**B**) Excitation and emission spectra of single Spinach aptamer (Spi) and Spinach arrays (100 nM RNA +20 μM DFHBI). (**C**) Fluorescence intensities of Spi and Spi-*n*R, measured by fluorometer.

We designed a series of Spinach arrays containing different numbers of tandem Spinach aptamers (Spi-*n*R, *n* = 8, 16, 32, 64). Between two adjacent aptamer repeats we inserted 17-nt randomized spacer sequence, which was used for constructing tandem repeats of MS2 RNA sequence^26^,^27^. The Spinach array was inserted into pET28a plasmid, placing both *in vitro* and cellular RNA expression under the control of T7 promoter and *lac* operator system, respectively^49^ (Fig. 1A). Fluorescence intensities of *in vitro* transcribed Spinach arrays (100 nM RNA) were measured using a fluorometer after RNA folding and incubation with DFHBI (20 μM). Compared to single Spinach aptamer (Spi), Spi-*n*R shows greatly enhanced fluorescence signal with the same maximum excitation/emission wavelengths at 460/505 nm (Fig. 1B). The fluorescence intensity increases with the repeat number: it changes approximately 16-fold when the aptamer repeat number increases 64-fold (Fig. 1C), with the average 1.6-fold fluorescence enhancement upon repeat number duplication (Supplementary Methods and Materials and Supplementary Fig. S1). Binding of DFHBI to aptamers within the array showed similar kinetics (i.e, *k*_on_, *k*_off_ and *K*_D_) as binding to single Spinach aptamer (Spi) (Supplementary Table S2).

There are two possible explanations for the less than 2-fold increase in the brightness when the repeat number was doubled: fluorescence quenching between adjacent aptamers, and misfolding/incomplete folding of multiple aptamers^27^,^31^. To test inter-aptamer quenching, we measured the fluorescence lifetimes of single Spinach and Spinach arrays. The average fluorescence lifetime of Spi, Spi-8R and Spi-32R was 4.0, 3.91 and 3.63 ns, respectively. Therefore, we estimated that quenching can account for only up to 10% in fluorescence reduction (Supplementary Methods and Materials). To test misfolding/incomplete folding, we used a previously reported assay to estimate the folding efficiency^35^,^50^ and found that the folding efficiency of Spi-8R relative to Spi is 43.5 ± 1.6% and drops further to 34.6 ± 2.0% for Spi-32R (Supplementary Table S1). Spinach is known to be subject to misfolding^50^ and Spi-*n*R may misfold more extensively through inter-aptamer misfolding, similar to the example where repeat proteins are known to misfold due to interdomain interactions^51^,^52^,^53^. Incorporating the single Spinach aptamer into a tRNA scaffold could slightly increase its fluorescence likely through improving the folding of the Spinach sequence (Supplementary Table S1 and Supplementary Fig. S2). However, recent study showed that the RNA aptamer incorporated into tRNA scaffold suffered endonucleolytic cleavage due to tRNA sequence recognition by RNases in bacteria and mammalian cells^54^, disfavoring the extensive use of tRNA scaffold in Spinach imaging.

**Figure 2:**
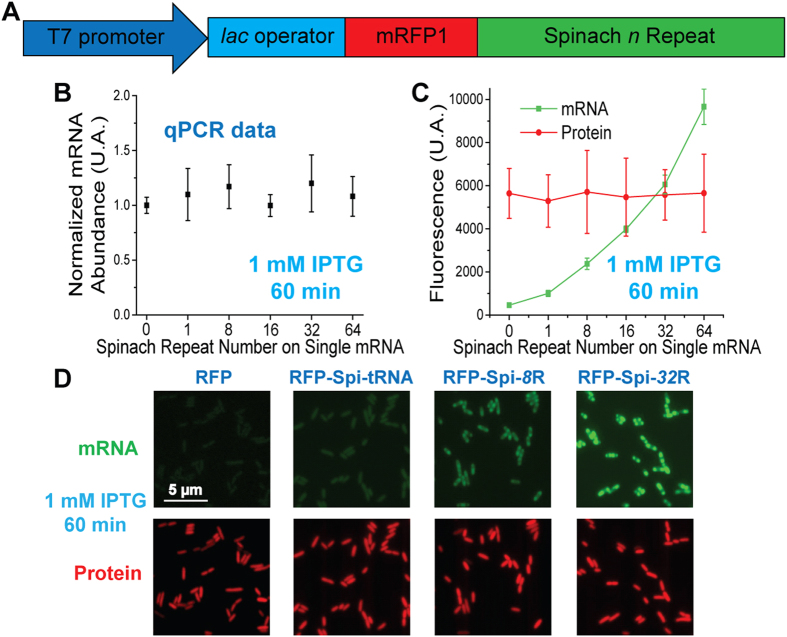
(**A**) Sketch of RFP-Spi-*n*R expression system in *E. coli*. (**B**) mRNA expression level of RFP or RFP-Spi-*n*R in *E. coli* after 60 min of 1 mM IPTG induction, measured by qPCR and normalized by the mRNA level of unmodified RFP in *E. coli*. (**C**) mRNA (Spinach) and protein (mRFP1) fluorescence in *E. coli* expressing RFP or RFP-Spi-*n*R upon induction, measured via epifluorescence imaging. (**D**) Representative fluorescence images of *E. coli* expressing RFP or RFP-Spi-*n*R upon induction.

In order to measure the fluorescence enhancement of Spinach array-tagged mRNA in living cells and characterize its influence on mRNA transcription, translation and degradation, we inserted Spinach arrays in the 3′ UTR (untranslated region) of the monomeric red fluorescent protein (mRFP1) coding sequence (henceforth called RFP-Spi-*n*R (*n* = 8, 16, 32, 64)) (Fig. 2A). For comparison, we also prepared two constructs each with a single Spinach: RFP-Spi, in which Spinach aptamer was directly linked to RFP sequence, and RFP-Spi-tRNA, in which Spinach aptamer was incorporated into a human tRNA^Lys^ scaffold as previously reported^34^,^55^. Quantitative reverse transcription PCR (qPCR) data showed that the cellular level of Spinach-tagged mRNA under induction (1 mM IPTG, 60 min), either by a single Spinach or by an array, is similar to that of untagged mRNA (Fig. 2B), suggesting that mRNA transcription is unperturbed by the Spinach tag even with the longest array tested. In addition, the average fluorescence intensities of mRFP1 protein per cell, translated from Spinach-tagged or untagged mRNAs, were comparable (Fig. 2C,D), indicating that mRNA translation is not affected by Spinach tag either as a single aptamer or as an array.

We then measured how much brighter Spinach arrays are compared to single Spinach in living cells. Live cell imaging of *E. coli* transcribing RFP-Spi showed no fluorescence enhancement in the Spinach channel over the background autofluorescence level of cells expressing untagged RFP (Supplementary Fig. S3). In comparison, cells transcribing RFP-Spi-tRNA showed homogenously distributed Spinach fluorescence with a slight enhancement over the autofluorescence level (Fig. 2C,D, and Supplementary Fig. S3). The fluorescence deficiency in cells transcribing RFP-Spi probably results from misfolding of the Spinach aptamer when fused to an mRNA, in line with the observation that *in vitro* transcribed and folded RFP-Spi did not show any Spinach fluorescence (Supplementary Fig. S2). In contrast, *E. coli* transcribing RFP-Spi-*n*R showed strong Spinach fluorescence. The increase in average fluorescence intensity per cell as a function of *n* (~17 fold enhancement from RFP-Spi-tRNA to RFP-Spi-64R, after autofluorescence subtraction) generally reflects the *in vitro* trend (~16 fold enhancement from Spi to Spi-64R). Moreover, distinct from the homogenously distributed fluorescence observed for RFP-Spi-tRNA, the fluorescence signal of RFP-Spi-*n*R preferentially accumulated at cell poles. In order to determine whether the apparent difference in cellular localization between the two constructs is caused by Spinach arrays, we conducted an RNA FISH experiment on cells expressing RFP, RFP-Spi-tRNA or RFP-Spi-*n*R (*n* = 8, 32), using Cy5-labeled oligonucleotide probes targeting the mRFP1 sequence (Supplementary Fig. S4) as well as the Spinach array (Supplementary Fig. S5). FISH data indicated that upon IPTG induction, mRNAs were preferentially accumulated at the cell poles where mRNAs transcribed from plasmid DNA are typically observed in *E. coli*^56^, regardless of the presence or the types of the Spinach tag at the 3′ UTR (Supplementary Fig. S4). Therefore, we conclude that the low fluorescence from single Spinach failed to reveal accurate mRNA localization (Fig. 2 and Supplementary Fig. S6) and the Spinach array could address the problem by greatly enhancing fluorescence signal for cellular mRNA imaging.

**Figure 3:**
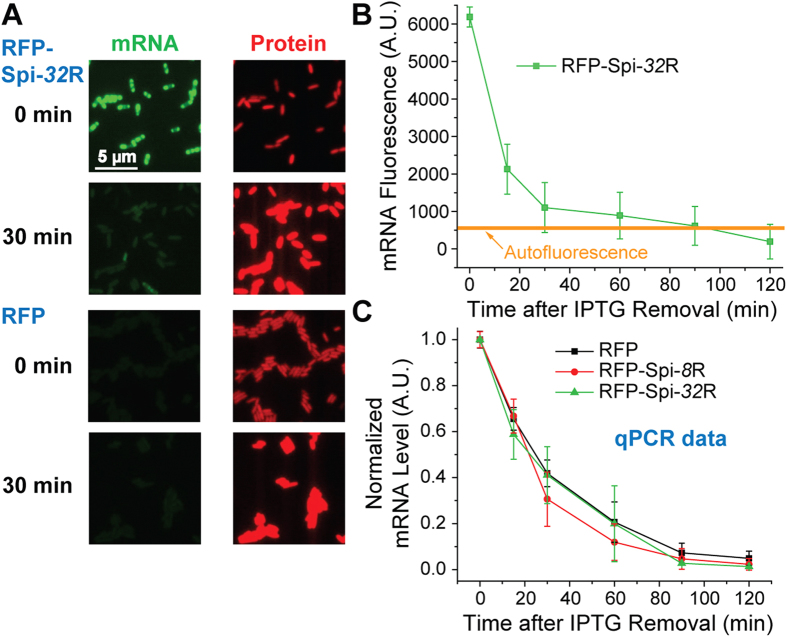
(**A**) mRNA (Spinach) and protein (mRFP1) fluorescence of *E. coli* expressing RFP-Spi-32R and RFP 0 min and 30 min after IPTG removal. (**B**) Spinach fluorescence as a function of time in the decay assay for RFP-Spi-32R, compared with the autofluorescence measured from *E. coli* expressing RFP. (**C**) RNA level in *E. coli* expressing RFP, RFP-Spi-8R and RFP-Spi-32R as a function of time in the decay assay, measured by qPCR.

Next, we examined the effect of Spinach arrays on RNA degradation. We conducted an mRNA decay assay for *E. coli* cells expressing RFP-Spi-32R or untagged RFP. We monitored the fluorescence of RFP-Spi-32R mRNA and mRFP1 protein after IPTG removal which would stop the synthesis of target RNAs. Spinach fluorescence levels greatly decreased within 30 min and were depleted within 90 min after withdrawal of IPTG suggesting mRNA degradation (Fig. 3A,B). We also compared mRFP1 fluorescence between *E. coli* expressing RFP-Spi-32R and untagged RFP (Fig. 3A and Supplementary Fig. S7). In both cases the fluorescence drop showed a significant time lag behind Spinach fluorescence change, which possibly resulted from the combination of the following: (1) continuous translation of mRFP1 from the remaining mRNA after IPTG removal, (2) much longer lifetime of protein compared to that of mRNA^57^,^58^,^59^, and/or (3) mRFP1 maturation^60^,^61^. We observed very similar trends of mRFP1 fluorescence intensity change in cells expressing RFP-Spi-32R and RFP (Supplementary Fig. S7), indicating that the Spinach array does not affect mRNA degradation or translation kinetics. To further confirm the effect of Spinach array on mRNA degradation, we conducted qPCR experiment to measure the abundance of untagged RFP mRNA and RFP-Spi-*n*R (n = 8, 32) as a function of time after IPTG removal (Fig. 3C). qPCR data showed that RFP-Spi-*n*R mRNA level was decreased by approximately 60% and 90% 30 and 120 min after IPTG removal, respectively, with the same trend observed for the untagged RFP. We therefore conclude that Spinach arrays do not influence mRNA decay.

**Figure 4:**
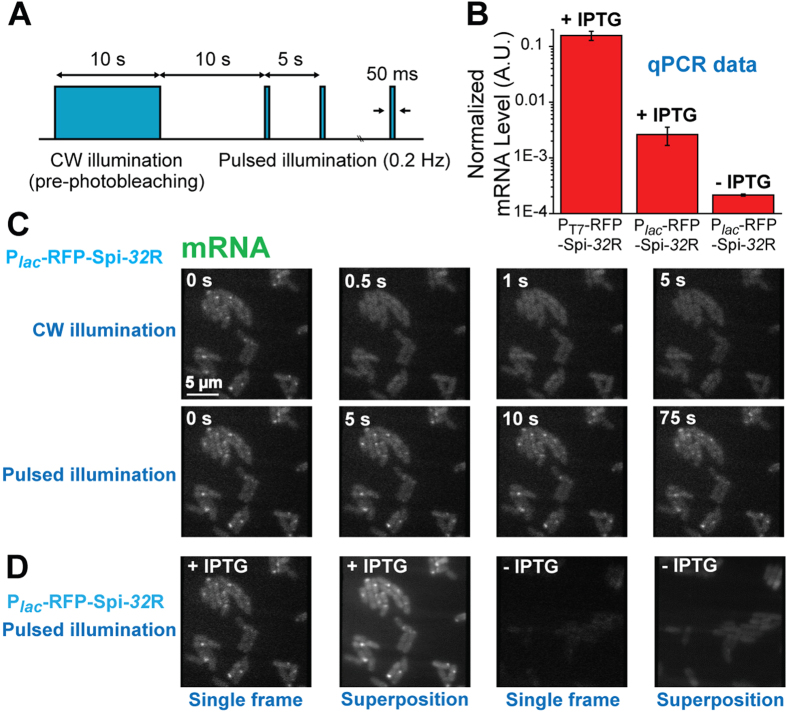
(**A**) The pulsed illumination strategy to observe Spinach fluorescence, in which a 10 s continuous-wave (CW) illumination was applied to pre-photobleach (pre-PB) cellular autofluorescence, with a 10 s wait period after illumination withdrawal, and then pulsed laser (power 0.2 mW, frequency 0.2 Hz, pulse duration 50 ms) was sent to illuminate the sample. (**B**) Expression level of RFP-Spi-*32*R mRNA under different promoters and induction conditions, measured by qPCR. (**C**) Representative fluorescence images of induced P_*lac*_-RFP-Spi-*32*R cells under CW or pulsed illumination. (**D**) Fluorescence images of induced and uninduced P_*lac*_-RFP-Spi-*32*R cells, shown in single frame (50 ms exposure time) or the superposition of 15 frames under pulsed illumination.

As the Spinach array effectively enhanced the fluorescence signal compared to single Spinach tag and performed well in quantitatively reporting the mRNA abundance in live cells through imaging, we then tuned the mRNA transcription level to further characterize the imaging sensitivity of the Spinach array. We replaced the T7 promoter (P_T7_-RFP-Spi-32R) with a native *lacZYA* promoter (P_*lac*_) for RFP-Spi-32R transcription (P_*lac*_-RFP-Spi-32R). RNA synthesis by endogenous *E. coli* RNA polymerase instead of T7 polymerase reduced the mRNA expression level by approximately two orders of magnitude according to qPCR quantification (Fig. 4B). Using 16S ribosomal RNA (16S rRNA) (~20,000–70,000 copies per *E. coli* cell^27^,^62^) as a reference, we estimated the copy number of RFP-Spi-32R mRNA transcribed under the control of P_*lac*_ and P_T7_ to be ~50–180 and ~3,000–11,000 per cell (Fig. 4B), respectively, which is consistent with the reported transcription levels for the two expression systems^63^,^64^. We first conducted epifluorescence microscopy on cells expressing P_*lac*_-RFP-Spi-32R with the same imaging condition applied for P_T7_-RFP-Spi-32R. P_*lac*_-RFP-Spi-32R did not show any fluorescence signal in Spinach channel beyond background autofluorescence level of uninduced P_*lac*_-RFP-Spi-32R (Supplementary Fig. S8), indicating that the epifluorescence microscope we used here is not sensitive enough to detect fluorescence signal from Spinach array with ~50–180 RNA copies per cell. In order to obtain higher fluorescence signal from the Spinach array, we used a 473 nm laser instead of the lamp light as the excitation source. A previous study showed that the DFHBI bound to the Spinach aptamer quickly dissociates (within ~100 ms) upon strong excitation, causing the loss of fluorescence^43^. To address the problem, we utilized pulsed excitation by laser to allow for Spinach to rebind a fluorogen after light-induced fluorogen dissociation and regain the fluorescence (Fig. 4A). Prior to imaging, we used continuous-wave (CW) illumination to decrease the cellular autofluorescence level (pre-photobeaching, or pre-PB) (Fig. 4A and Supplementary Fig. S9)^65^. Afterwards we used an automatically controlled mechanical shutter to generate a 0.2 Hz repetitive laser pulse with 50 ms pulse width^43^ (Supplementary Methods and Materials). With the help of pulsed illumination, we could observe clear distinction between uninduced P_*lac*_-RFP-Spi-32R (~4–15 mRNA copies per *E. coli* cell), where we hardly observed any fluorescence signal (Fig. 4D), and induced P_*lac*_-RFP-Spi-32R (~50–180 mRNA copies per *E. coli* cell), where we found bright spots in many cells residing at cell poles (Fig. 4C,D). We attribute the bright spots to transcription sites containing multiple mRNAs. The bright spots disappeared within a very short illumination time (<500 ms) and reoccurred upon illumination withdrawal (>5 s) followed by reinstatement (Fig. 4C), which is a typical optical characteristics of Spinach fluorescence^43^, suggesting that the fluorescence originated from the Spinach array. If we superposed multiple cell images from repeated cycles to achieve stronger fluorescence, we could clearly find preferential localization of the fluorescence at cell poles (Fig. 4D), consistent with the observed mRNA localization of induced P_lac_-RFP-Spi-32R verified by RNA FISH (Supplementary Fig. S10). Overall, with the help of the pulsed illumination method, we further enhanced the performance of the Spinach array and showed the potential to apply the system to image lower abundance cellular RNA.

## Discussion

The Spinach system is a recently developed RNA labeling and imaging method based on aptamer binding and fluorescence induction of the fluorogenic small molecule DFHBI^34^. It has many potential advantages over widely applied RNA labeling methods using fluorescent protein-fused RNA binding proteins, such as low fluorescence background, elimination of separate introduction of RNA binding proteins, and evasion of perturbation on target RNAs by protein binding. However, there were few reports on single Spinach aptamer labeling for cellular mRNA imaging^42^, likely due to its low fluorescence brightness suggested by our experimental data (Fig. 2D). To address this problem, we employed a tandem Spinach array to tag a single mRNA molecule and demonstrated that the Spinach array containing 64 aptamer repeats can enhance the fluorescence by 17-fold in living cells compared to a single Spinach aptamer. The fluorescence enhancement by the Spinach array greatly improved mRNA imaging quality compared to using the single Spinach tag. In particular, we observed inhomogenous RNA distribution and distinct RNA loci in E. *coli* (Fig. 3D and Supplementary Fig. S4) using the Spinach array, whereas single Spinach tag suffered from low fluorescence signal and failed to report correct RNA localizations in cells. For cells with a lower mRNA level (~120–180 mRNAs per E. *coli*.), we further applied pulsed illumination strategy to effectively boost the fluorescence of the Spinach array such that we could observe mRNA localizations. Despite the 17-fold fluorescence enhancement achieved by constructing the aptamer array, we also noted that the average efficiency for an aptamer to correctly fold seems to decrease with the increase of the aptamer repeat number. One possible reason for this is the crosstalk and mispairing between adjacent or spatially close aptamers in a tandem array, which were reported and discussed for tandem arrays in previous RNA and protein folding studies^51^,^52^,^53^,^66^. In addition, the intrinsic instability of the Spinach aptamer may also play a role (~32% folded, 25 °C^35^). Future introduction of several recently reported aptamers with improved folding efficiency (Spinach 2, ~58%^35^; Broccoli, ~60%^50^) may enhance the fluorescence of the aptamer array. It is also possible that optimizing linker sequences may improve the performance although mispairing-based misfolding may not be avoided by changing linker sequences alone.

With a series of characterizations on the aptamer tandem array, we demonstrated that in *E. coli*, Spinach array-tagged mRNA had no significant alterations on transcription, translation or degradation. This may be attributed to the small size and high dissociation constant (*K*_*D*_) of the fluorogen. In contrast, the MS2/PP7 coat protein labeling method has been reported to impede RNA degradation in bacteria in previous studiess^26^,^27^, likely due to the stable association of many MS2/PP7 proteins to mRNA, which prevents the bacterial RNA degradation machinery from functioning. In addition, the Spinach array did not alter mRNA localization. When applied to eukaryotic cell imaging, in order to decrease the fluorescence background introduced by the MS2 coat protein-fluorescent protein fusion (MS2-FP) in cytosol, the MS2-FP proteins are usually fused with a nuclear localization sequence (NLS) to guide excess unbound proteins to the nucleus^11^,^48^. Although the strategy increases the signal-to-noise ratio, it might potentially perturb the endogenous RNA localization. In contrast, DFHBI remains non-fluorescent until binding to the Spinach aptamer, which circumvents extra modifications that decrease background fluorescence but could possibly affect target RNA localization.

In conclusion, we constructed a tandem Spinach aptamer array that could enhance fluorescence imaging quality of the Spinach/DFHBI system for live cell RNA imaging while introducing minimal perturbation to the target RNA. Nevertheless, Spinach arrays still show several limitations, including relatively low brightness and large size, making them ill-suited for studying mammalian cells, where autofluorescence is stronger and RNAs are more dispersedly distributed and undergo intensive motor-driven transport. Several recently developed fluorogen/aptamer systems, including RNA Mango, Spinach 2, Broccoli, *etc*, showed the potential to further enhance the performance. All of those newly discovered fluorogen/aptamer systems have similar fluorescing mechanisms as that of the Spinach aptamer while showing higher brightness, shorter length or more robust folding behavior. We envision that incorporating the newly reported aptamers into a tandem array could also enhance their fluorescence signals per RNA molecule. Furthermore, we expect the tandem arrays derived from those aptamers would be very likely to bring minimal perturbation to their target RNAs, due to their similarities to the Spinach aptamer.

## Methods

The methods can be found in the Supplementary Information.

## Additional Information

**How to cite this article**: Zhang, J. *et al.* Tandem Spinach Array for mRNA Imaging in Living Bacterial Cells. *Sci. Rep.* **5**, 17295; doi: 10.1038/srep17295 (2015).

## Supplementary Material

Supplementary Information
