# Supplementary material for: Tandem Spinach Array for mRNA Imaging in Living Bacterial Cells
Source: Sci Rep. 2015 Nov 27;5:17295. doi: 10.1038/srep17295 (PMC4661537; doi:10.1038/srep17295)
Supplement: Supplementary Information [file srep17295-s1.pdf]

Supplementary Information

Tandem "Spinach" Array for mRNA Imaging in Living Bacterial Cells

Jichuan Zhang[1,2], Jingyi Fei[1], Benjamin J. Leslie[1,5], Kyu Young Han[1,5], Thomas E. Kuhlman[1], Taekjip Ha[1,3,4,5]*

[1]Department of Physics, University of Illinois at Urbana-Champaign, Urbana, IL 61801 USA.
[2]Department of Materials Science and Engineering, University of Illinois at Urbana-Champaign, Urbana, IL 61801 USA.
[3]Department of Biophysics and Biophysical Chemistry, Johns Hopkins University, Baltimore, MD 21205 USA.
[4]Department of Biomedical Engineering, Johns Hopkins University, Baltimore, MD 21205 USA.
[5]Howard Hughes Medical Institute, Urbana, IL 61801 USA.
*corresponding. tjha@jhu.edu

Methods and Materials

Chemical synthesis of DFHBI

DFHBI was synthesized according to the procedure introduced by Paige and coworkers[1], and was dissolved in DMSO to 10 mM final concentration and stored at -20 °C.

Spi-tRNA and Spi sequence

Spi-tRNA, containing one repeat of the 24-2 aptamer inside a $tRNA^{Lys}$ scaffold in the pET28c vector was given as a kind gift by Prof. Samie Jaffrey (Weill Cornell Medical College)[1]. The 24-2 sequence was amplified by PCR from pET28c-Spi-tRNA and inserted into pET28a plasmid (Novagen) between XbaI and HindIII restriction sites to create plasmid Spi-1R. Plasmids were transformed into BL21-DH5α (Promega) and BL21-DE3-Rosetta (Novagen) *E. coli* strains for DNA cloning and live cell imaging, respectively. Please see Supporting Information for complete sequence information.

Design and construction of Spinach arrays and RFP-Spi, -Spi-tRNA and -Spi-*n*R

The Spi-*8*R sequence was synthesized by Genscript as double-stranded DNA and was delivered in the pUC57 plasmid (Genscript) between the EcoRI and HindIII restriction sites (pUC57-Spi-*8*R). Each repeat sequence contains the Spinach (minimal) sequence, or 24-2-min sequence reported by Paige and coworkers[1], plus a 17 nt randomized linker sequence. The Spi-8R was then excised with XbaI and HindIII and inserted into pET28a plasmid between the XbaI and HindIII restriction sites to generate pET28a-Spi-*8*R.

The repeat expansion method to construct Spi-*16*R, -*32*R and -*64*R was previously described by Golding and coworkers and exploits differences in recognition sequences of the isocaudamers XbaI and NheI[2]. Briefly, we digested the pET28a-Spi-*8*R plasmid in two separate reactions- XbaI and HindIII were used to generate the insert fragment Spi-*8*R, which was inserted into the pET28a-Spi-*8*R backbone digested with NheI and HindIII, to obtain pET28a-Spi-*16*R. The cycle was repeated to create pET28a-Spi-*32*R and -*64*R.

The mRFP1-coding sequence was amplified by PCR from pTRUEBLUE-BAC2-$P_{lac/ara}$-mRFP1-96BS constructed by Golding and coworkers[3] , and was inserted into pET28a between NcoI and NdeI to generate pET28a-RFP plasmid. The further construction of pET28a-RFP-Spi, -Spi-tRNA, -Spi-*n*R plasmid was accomplished by inserting the Spinach sequence flanking XbaI and HindIII sticky ends into pET28a-RFP plasmid between NheI and HindIII restriction sites.

To replace T7 promoter in pET28a-RFP-Spi-32R plasmid, a *lacZYA* (*lac*) promoter-*lac* operator sequence, amplified by PCR from pUC57-Spi-*8*R (Genscript), flanking BglII and XbaI sticky ends was inserted into the plasmid between BglII and XbaI restriction sites. Please see Supporting Information for complete sequence information.

In vitro transcription and RNA folding

Spi and Spi-*n*R (n = 8, 16, 32, 64) RNA were synthesized by *in vitro* transcription using MEGAshortscript T7 kit (Life Technologies) and MEGAscript T7 Kit (Life Technologies), respectively, following the protocol. The transcribed RNA was buffer-exchanged twice with a P-6 micro bio-spin column (Bio-Rad) into RNA storage buffer (10 mM Tris acetate (pH 8.0), 0.1 mM EDTA and 10 mM KCl) to remove unreacted nucleotides. The RNA was folded in selection buffer (40 mM K-HEPES (pH 7.5) and 125mM KCl[1]) by incubation at 90 °C in water bath for 2 min, followed by slow cooling down to 65 °C, and then supplemented with 5 mM $MgCl_2$ to assist RNA folding and further cooling down to room temperature.

Fluorometer measurement of *in vitro* transcribed RNA

Folded Spi-tRNA and Spi-*n*R (*n* = 8, 16, 32, 64) RNA was diluted in selection buffer (40 mM K-HEPES (pH 7.5), 125 mM KCl) supplemented with 5 mM $MgCl_2$ and incubated with 20 µM DFHBI at room temperature (22 °C) for 5 min. Fluorescence measurement of the RNA/DFHBI complex was performed with a fluorometer (Cary Eclipse Fluorescence Spectrophotometer, Agilent Technologies) using the following instrument parameters: "Scan" mode; excitation wavelength 460 nm, excitation slit width 5 nm; emission wavelength 480 - 600 nm, emission slit width 5 nm.

To obtain the excitation and emission spectrum of Spi-tRNA, Spi and Spi-*n*R RNA, the following instrument parameters were applied: (1) excitation spectrum, emission wavelength 505 nm and emission slit width 5 nm, excitation wavelength 400 - 480 nm and excitation slit width 5 nm; (2) emission spectrum, excitation wavelength 460 nm and excitation slit width 5 nm, emission wavelength 480 - 580 nm and emission slit width 5 nm.

Fluorescence enhancement efficiency measurement

We defined the fluorescence enhancement efficiency as the fluorescence intensity increment when the aptamer repeat number is doubled in the Spinach array. The value of the enhancement efficiency is located between 0, when the fluorescence intensity remains unchanged, and 1, when the fluorescence intensity is doubled, upon aptamer number plication. The fluorescence intensity of Spi-*n*R, $I_{Spi\text{-}nR}$, as a function of the enhancement efficiency can be formulated as:

$$I_{Spi\text{-}nR} = I_{Spi} \times (1+\text{eff})^{\log 2(n)}$$

In the equation $I_{Spi\text{-}nR}$ is the *in-vitro* fluorescence of Spi-*n*R (Figure 1C), I_{Spi} is the *in-vitro* fluorescence of Spi (Figure 1C), "eff" is the enhancement efficiency, and $\log_2(n)$ is the value of logarithms base 2 of *n*, which we further defined as duplication round, or the number of repeat duplication required to construct Spi-*n*R from Spi. We can transform the formula into:

$$\log_2(I_{Spi\text{-}nR}) = \log_2(1+\text{eff}) \times \log_2(n) + \log_2(I_{Spi})$$

The enhancement efficiency value can be obtained by plotting $\log_2(I_{Spi\text{-}nR})$ as a function of $\log_2(n)$ and conducting linear fitting to obtain the slope value, which is the $\log_2(1+\text{eff})$ in the equation. Then the enhancement efficiency can be calculated with the slope value. We put the data plot and linear fitting in Supplementary Figure S1, and had the slope value 0.67 and the fluorescence enhancement efficiency value 0.59.

Relative aptamer folding efficiency measurement of Spi-*n*R compared to Spi

The measure the relative folding efficiencies of Spi, Spi-8R and Spi-32R compared to Spi-tRNA, we kept the aptamer amount identical for Spi-tRNA, Spi, Spi-8R and Spi-32R, and incubated the RNA with excess amount of DFHBI. The solution was sent for fluorescence measurement and the fluorescence of Spi, Spi-8R and Spi-32R was compared with that of Spi-tRNA to calculate the relative folding efficiencies of Spi, Spi-8R and Spi-32R compared to Spi-tRNA. In the experiment we incubated 100 nM Spi-tRNA, 100 nM Spi, 12.5 nM Spi-8R or 3.125 nM Spi-32R with 10 µM DFHBI, respectively, making the aptamer concentration in each solution 100 nM and DFHBI 100 times the concentration of the aptamer.

Spinach binding kinetics measurement

The fluorescence increase was recorded with a fluorometer (Cary Eclipse Fluorescence Spectrophotometer, Agilent Technologies) after rapid mixing of certain concentration of Spinach RNA (40 nM Spi, 10 nM Spi-8R or 5 nM Spi-32R) with different concentrations of DFHBI (200 - 700 nM) in a cuvette (100 µL). The excitation and emission wavelengths were 460 and 505 nm, respectively. The excitation and emission slit widths were 5 nm and 20 nm, respectively. The illumination intensity was estimated to be 1–5 W/cm2.

The Spinach and DFHBI binding reaction is described with a simple kinetic model as below:

$$\text{Spinach} + \text{DFHBI} \rightleftharpoons \text{Spinach} - \text{DFHBI}$$

$$\frac{d[\text{Spinach} - \text{DFHBI}]}{dt} = \text{kon} \times [\text{Spinach}] \times [\text{DFHBI}] - \text{koff} \times [\text{Spinach} - \text{DFHBI}]$$

$$\frac{d[\text{Spinach}]}{dt} = \frac{d[\text{DFHBI}]}{dt} = \text{koff} \times [\text{Spinach} - \text{DFHBI}] - \text{kon} \times [\text{Spinach}] \times [\text{DFHBI}]$$

In order to directly compare the DFHBI binding kinetics between single Spinach aptamer (Spi, single binding site) and tandem Spinach arrays (Spi-*n*R, multiple binding sites), we estimate the effective concentration of functional Spinach aptamers within tandem Spinach arrays for our data analysis, considering the relative folding efficiency of tandem Spinach arrays (43.5% for Spi-*8*R and 34.6% for Spi-*32*R; Supplementary Table S1) we measured separately. The effective initial concentrations of the RNA and DFHBI in the experiments were: Spi, 40 nM, 40 nM Spinach aptamer; Spi-*8*R, 10 nM, 34.8 nM Spinach aptamer; Spi-*32*R, 5 nM, 55.4 nM Spinach aptamer; DFHBI, 200 - 700 nM.

We obtained the concentration of Spinach/DFHBI binding complex as a function of time (Supplementary Figure S11). We used MATLAB to simulate the time-course change of the complex concentration, and fit the simulated curve to our experimental data to determine k_{on} and k_{off} by maximizing R^2: k_{on}, k_{off} and K_{D} values are all reported in Supplementary Table S2.The fluorescence intensity of Spi, Spi-8R and Spi-32R all displayed a biphasic behavior (Supplementary Figure S11), a fast increase followed by a much slower increase. We hypothesized that additional conformational change of the aptamer resulted in a second slower phase with potentially altered k_{on} and k_{off}. However, with the single observable in our experiment, we cannot determine the parameters for the additional kinetic steps. Therefore, we focused on the fast phase, and simulated the first 100 s of the binding curve and determined the respective k_{on}, k_{off} and K_D value of DFHBI binding onto Spi, Spi-8R and Spi-32R under various DFHBI concentration conditions (Supplementary Figure S12A). With the determined k_{on} and k_{off} values, we predicted a smaller value than the experimentally measured value at the plateau region. Nevertheless, the predicted value can capture 85% of the experimental value for Spi (Supplementary Figure S12B), and 80% for Spi-*8*R and Spi-*32*R.

We also found that for Spi, Spi-8R and Spi-32R, the k_{on}, k_{off} and K_D value were consistent at various DFHBI concentrations (200 - 700 nM). The average value of the k_{on} and k_{off} we calculated (Spi, $k_{on} = (8.3 \pm 1.1)\times 10^4$ $M^{-1}\cdot s^{-1}$, $k_{off} = (2.5 \pm 0.4)\times 10^{-2}$ s^{-1}; Spi-*8*R, $k_{on} = (9.3 \pm 0.6)\times 10^4$ $M^{-1}\cdot s^{-1}$, $k_{off} = (2.3 \pm 0.7)\times 10^{-2}$ s^{-1}; Spi-*32*R, $k_{on} = (7.3 \pm 1.1)\times 10^4$ $M^{-1}\cdot s^{-1}$, $k_{off} = (3.0 \pm 0.5)\times 10^{-2}$ s^{-1}) also indicated that the binding kinetics between DFHBI and the Spinach aptamer did not change significantly from single Spinach aptamer (Spi) to tandem Spinach arrays (Spi-*8*R and Spi-*32*R).

Time-resolved fluorescence measurement and fluorescence lifetime calculation

The fluorescence lifetime measurements were performed by a custom built confocal microscope as described elsewhere in details [Han et al., JACS (2013)]. An excitation light (473 ± 5 nm; ~ 10 ps pulse duration) was generated from an ultrafast laser (MaiTai HP, Spectra Physics) with the use of a photonic crystal fiber (FemtoWhite 800, NKT Photonics) and a pre-stretcher (N-SF57, CASIX). The fluorescence signal was detected by an avalanche photo diode (Micro Photon Devices) and registered by a time correlated single photon counting module (SPC630, Becker Hickl).

The extent of quenching (η) of Spi-*n*R compared with Spi-tRNA was calculated as follows. η = 1 - QY(Spi-*n*R)/QY(Spi-tRNA) = 1- τ f(Spi-tRNA)/ τ f(Spi-*n*R), where QY and τ f denote quantum yield and fluorescence lifetime, respectively.

Bacteria growth and induction

E. coli cells were grown at 37 °C with antibiotics according to the plasmid selection markers (100 μg/mL ampicillin (Gold Biotechnology, Inc) for pUC57 and pUC57-Simple plasmid, 50 μg/mL Kanamycin (Roche Diagnostics) for pET28a and pET28c plasmid, 30 μg/mL Chloramphenicol (Sigma-Aldrich) for pTRUEBLUE-BAC2 plasmid, and 30 μg/mL Chloramphenicol for *E. coli* Rosetta strain) in Lysogeny Broth (LB) (LB Broth Miller, EMD Millipore) liquid and solid media. To gauge cell density, optical density (OD) of the medium was assayed at 600 nm using a plastic cuvette in a Spectramax Plus 384 Microplate Reader (Molecular Devices, Inc.). For RNA and protein induction, Rosetta cells transformed with a given plasmid were grown at 37 °C in LB medium overnight from a single colony, and diluted 1000-fold into fresh LB medium and kept growing until at $OD_{600} = 0.2$. IPTG (Sigma-Aldrich) was then supplemented with 1 mM final concentration to induce RNA and protein production under T7 promoter-*lac* operator control. To maintain exponential growth of the cells, pre-warmed medium was added to dilute the cell culture to $OD_{600} = 0.3$ whenever OD_{600} of the culture exceeded 0.5.

RNA decay assay

Cells were grown in LB medium and induced by IPTG as described above. After 60 min induction by 1 mM IPTG, the cells were centrifuged to a pellet and the supernatant was carefully aspirated and the cell pellet was resuspended in fresh pre-warmed LB medium without IPTG. The centrifugation and resuspension process was performed twice to remove the remaining IPTG, and the cell pellet was finally resuspended in LB medium without IPTG. The cells were grown at 37 °C, and at different time points after IPTG removal, a bit of cell culture was taken out for imaging.

Epifluorescence microscopy and image analysis

To prepare the imaging sample, 1 mL of cell culture was supplemented with DFHBI to 100 μM final concentration 10 min before imaging, and was kept growing at 37 °C for DFHBI permeation and binding to Spinach aptamer. The cells were then centrifuged and supernatant was removed. After cell resuspension in pre-warmed M9 minimal medium (M9, Minimal Salts, 5X; Sigma-Aldrich) supplemented with 2mM $MgCl_2$ and 100 μM DFHBI, a few μL were sandwiched between a glass coverslip (No. 1.5) and a thin slab of 1.5 % (w/v) agarose gel. M9 minimum medium containing 2mM $MgCl_2$ and 100 μM DFHBI or 1x PBS medium was used to dissolve the agarose and make the gel for live cell imaging or FISH imaging, respectively.

All epifluorescence images were taken with a Nikon Eclipse (TE-2000-U, Nikon) microscope equipped an oil immersion objective (1.3 NA 100x) and an epifluorescence system. The epifluorescence system used a lamp light source (X-Cite Series 1200, Excelitas Technologies) to illuminate the sample and an emCCD camera (iXon3 897, Andor Technology) to acquire the fluorescence image. The filter sets applied were: Brightfield (exposure time 100 ms), Spinach (Ex 450-490 nm, Em 500-550 nm, exposure time 200 ms), mRFP1 protein (Ex 540-580 nm, Em 593-668 nm, exposure time 10 ms), Cy5 FISH probe (Ex 590-650 nm, Em 663-738 nm, exposure time 100 ms). The images were processed and analyzed using a MATLAB code reported by Golding and coworkers[3], which is able to identify single cells and measure the average fluorescence level of each cell in different fluorescence channels.

Pulsed illumination microscopy and image analysis

Pulsed illumination imaging was conducted by a home-built objective-TIRF microscope with an oil immersion objective (1.4 NA 100x, Olympus) equipped with an emCCD camera (iXon DU-887, Andor Technology). Illumination with a 473 nm laser (MLL-III-473, Opto Engine LLC) was controlled by a mechanical shutter (UniBlitz VMM-D3) through a National Instruments NI-6503 digital I/O controller card and synchronized to CCD via home-built software (cplc.illinois.edu/software)[4], and the data acquisition and analysis procedures conducted. The recorded movie was processed by a MATLAB code to generate a collection of fluorescence images of consecutive single frames. The superposed image was conducted by stacking selected frames in ImageJ.

Total RNA extraction and purification

For each sample, the cell culture was measured OD_{600}. We took out appropriate volume of the cell culture which contained the same total cell number of 1 mL 0.5 OD_{600} cells. The cells were centrifuged at 5,000 g and 4 °C for 5 min, and the supernatant was carefully aspirated. The cell pellet was lysed by 1 mM lysozyme/TE buffer (10 mM Tris:HCl, 1 mM EDTA, pH = 8.0) and total RNA was extracted and purified from the cell lysate through RNeasy Mini Kit (Qiagen) according to the protocol. 10 μg extracted RNA were further treated with DNase using Turbo DNA-free kit (Life Technologies) to remove remaining DNA which interfered with qPCR experiments. Afterwards reverse transcription reaction was conducted with 100 ng RNA in a 20 μL reaction volume to synthesize cDNA required for qRT-PCR experiments using iScript cDNA Synthesis Kit (Bio-Rad) from the DNAse-treated RNA, according to the protocol.

Quantitative reverse transcription PCR (qPCR) and transcript number estimation

1 μL reverse-transcribed cDNA were taken out of the 20 μL total volume of each reverse transcription reaction, and were diluted to 10 μL. Regular PCR reactions were first conducted using the diluted cDNA and designed qPCR primers to confirm that proper cDNA products were generated. After that 1 μL of the diluted cDNA were supplemented with qPCR primer and reaction and detection SsoAdvanced SYBR Green Supermix (Bio-Rad) to a 20 μL reaction volume and qPCR reactions were assembled in a 96-well PCR plate (Bio-Rad). qPCR primers targeting the mRFP1-coding sequence were used to quantify RFP-Spi-*n*R RNA, and primers targeting 16S ribosomal RNA were used to quantify 16S ribosomal RNA

as the internal standard. The qPCR reactions were conducted and monitored by Bio-Rad CFX96 Touch Real-Time PCR Detection System.

The expression level and the cellular transcript number of RFP or RFP-Spi-*n*R mRNA were roughly estimated by calculating the relative RNA expression level compared to that of 16S rRNA, using ΔC_T Method[5]. We have made a series of known dilutions of the cDNA samples and created standard curves of mRFP1 and 16S rRNA qPCR primers by plotting the threshold-crossing cycle number (C_T) of the amplification curves, to estimate the amplification efficiencies of both primer pairs, and to confirm that their amplification efficiencies are similar and reliable (between 90% and 105%). The relative expression level of a specific sample between RFP (-Spi-*n*R) mRNA compared to that of 16S rRNA can be calculated simply with the primer amplification efficiencies extrapolated from the standard curves and the C_T values measured by the qPCR experiments. The approximate RNA copy numbers per cell for RFP (-Spi-*n*R) mRNA were estimated by translating the relative value into absolute copy number using the value of 20,000 - 70,000 16S rRNA molecules reported by previous study[3,6,7].

RNA fluorescence *in situ* hybridization (FISH)

The FISH probes, which are DNA oligonucleotides with 3' amine modification, were designed and ordered from Biosearch Technologies, and combined and labeled with Cy5 NHS (GE Healthcare). The protocol of RNA FISH, including probe design and labeling, cell fixation and permeabilization, and probe hybridization, were reported by So and coworkers[8,9]. Cell preparation and sample hybridization are briefly described below.

After cell harvest (equivalent to 15 mL OD_{600} = 0.4) and centrifugation (4 °C, 4500x *g*, 8 min), supernatant was removed and cells were resuspended in 1 mL freshly prepared fixation solution (1x PBS, 3.7% (w/w) formaldehyde) and gently shaken at room temperature for 30 min. Centrifuge (400x *g*, 8 min) the cell suspension, remove supernatant, and then wash twice in 1 mL 1x PBS. Resuspend the cells in 70% (w/v) Ethanol, and leave the cell suspension at room temperature for at least 1h to permeate the cell. Afterwards centrifuge (600x *g*, 7 min) and remove supernatant. Resuspend cells in 1 mL 10% wash buffer (10% formamide (v/v), 2x SSC) and leave at room temperature for a few minutes, and then centrifuge (600x *g*, 7 min) and remove supernatant. Resuspend the cells well in 50 µL hybridization solution, which is supplemented with proper amount of Cy5-labeled combined probes (15 ng for each probe binding sites according to the target RNA, i.e. 13 binding sites for RFP sequence, 24 binding sites for Spi-*8*R and 96 binding sites for Spi-*32*R) and 50 µL 10% hybridization buffer (10% dextran sulface (w/v), 10% formamide (v/v), 1mg/mL *E. coli* tRNA, 2x SSC, 0.2 mg/mL BSA, 2 mM Ribonucleoside Vanadyl Complex), and leave at 30 °C overnight. On the next day, take a few µL of hybridization sample, add 20 volumes of 10% wash buffer, followed by mixing and centrifugation (600x *g*, 7 min). Afterwards repeat the following steps 3 times: resuspend the cells with 20 volumes 10% wash buffer after removing supernatant, incubate for 30 min at 30 °C, and centrifuge and remove supernatant. Finally resuspend the cells in 1 volume of 2x SSC and the cell resuspension are ready to image. Prepare 1.5% (w/v) agarose/PBS gel for imaging sample preparation, as described before. Cell sample was imaged using epifluorescence microscopy with the filter set mentioned above for Cy5 FISH probe.

Supplementary Figures

Figure S1

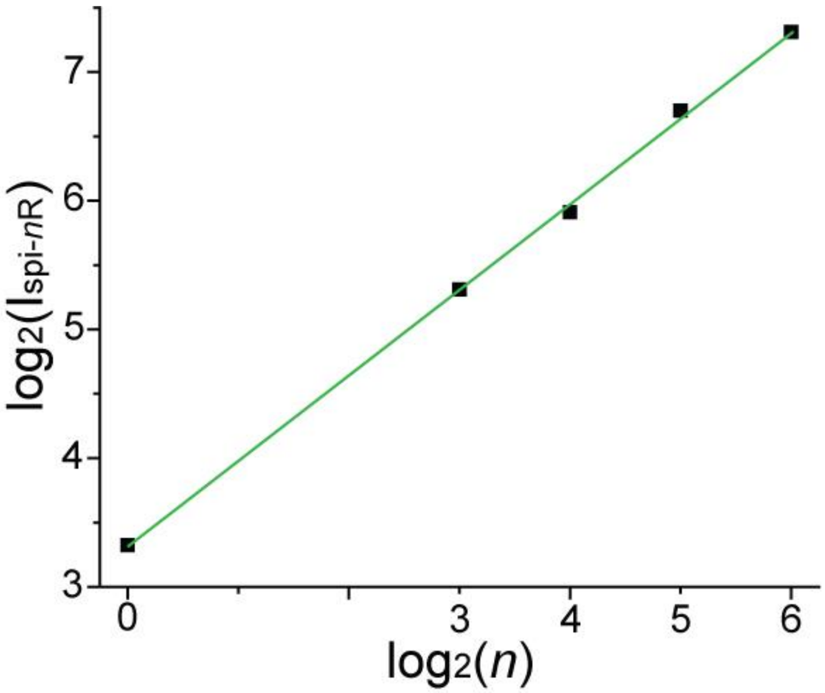

Figure S1. The plot of the *in-vitro* Spi-*n*R ($I_{Spi\text{-}nR}$) fluorescence intensity as a function of the aptamer repeat number (*n*), after taking base 2 logarithm of both the fluorescence intensity and the repeat number value. The green line is the linear fitting of the data. The slope of the fitting curve, which is 0.67 here, is used to calculate the fluorescence enhancement efficiency value, which is used to describe the *in-vitro* fluorescence fold enhancement upon aptamer repeat number duplication within the Spinach array. The calculated efficiency value is 0.59 here.

Figure S2

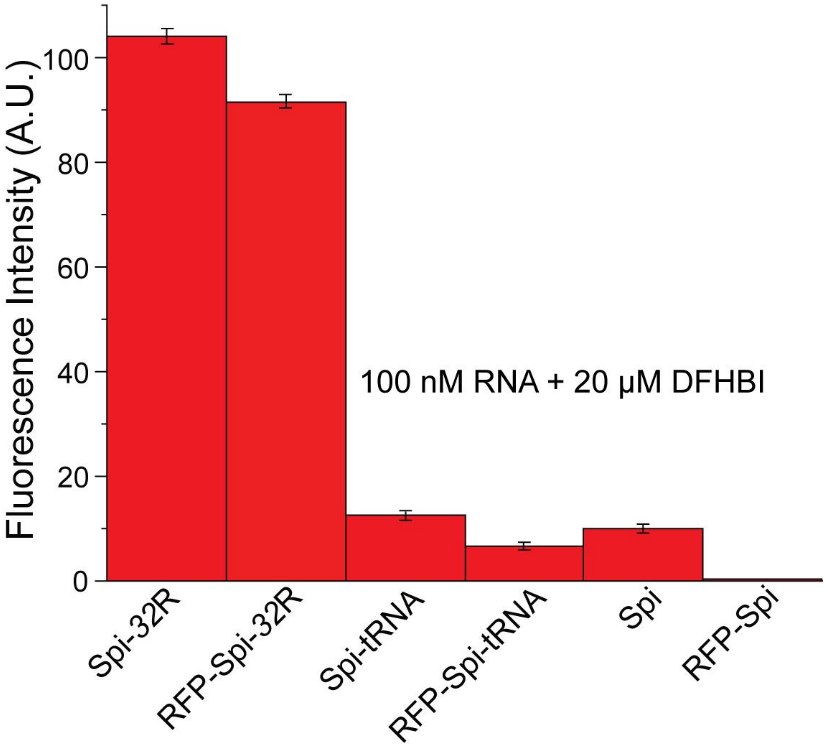

Figure S2. Fluorescence intensity of in vitro transcribed single Spinach (Spi, Spi-tRNA, RFP-Spi, RFP-Spi-tRNA) and Spinach arrays (Spi-32R, RFP-Spi-32R) in the absence or presence of adjacent mRFP1-coding sequence, measured by fluorometer.

Figure S3

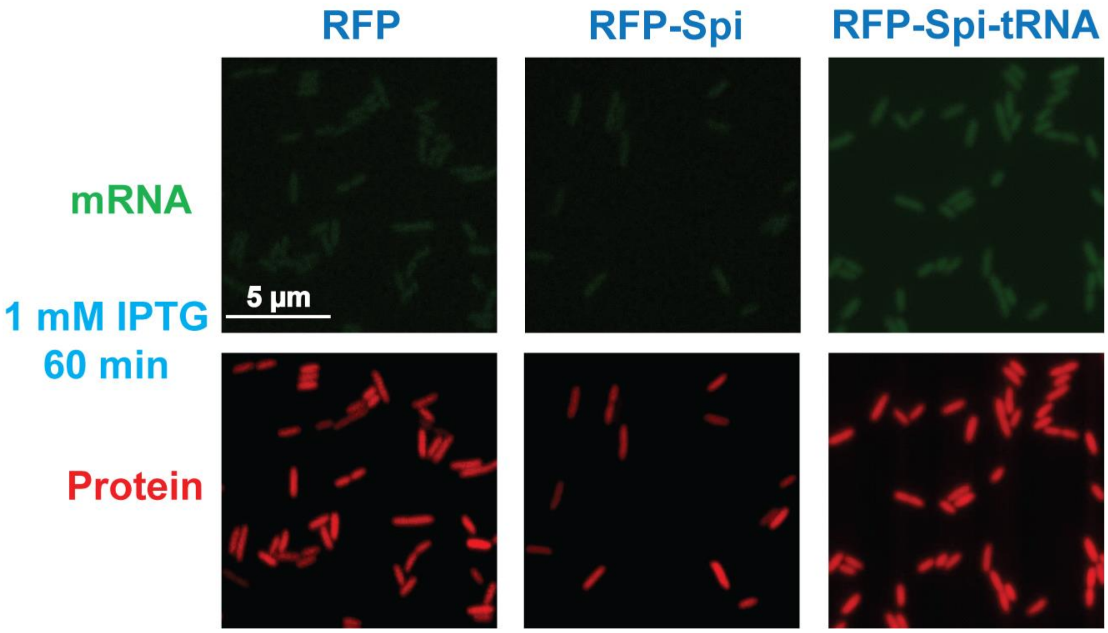

Figure S3. Representative fluorescence images of *E. coli* expressing RFP, RFP-Spi and RFP-Spi-tRNA

Figure S4

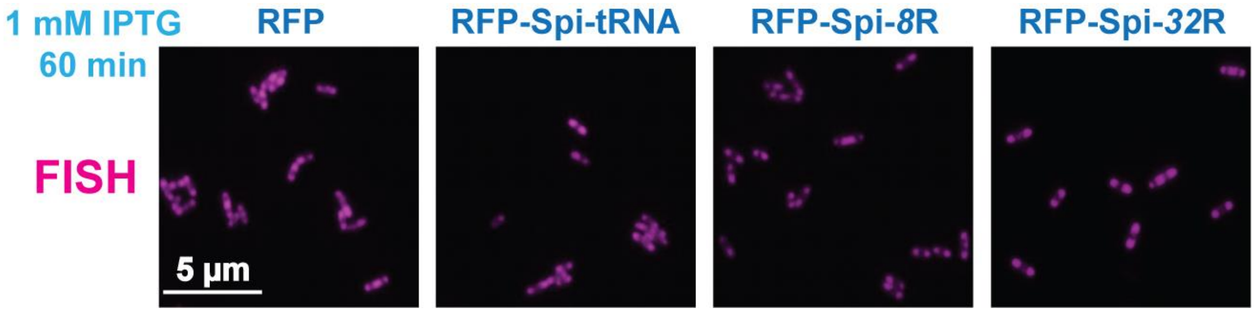

Figure S4. FISH validation of the localization of Spinach-tagged RFP mRNA, compared to that of unmodified RFP mRNA.

Figure S5

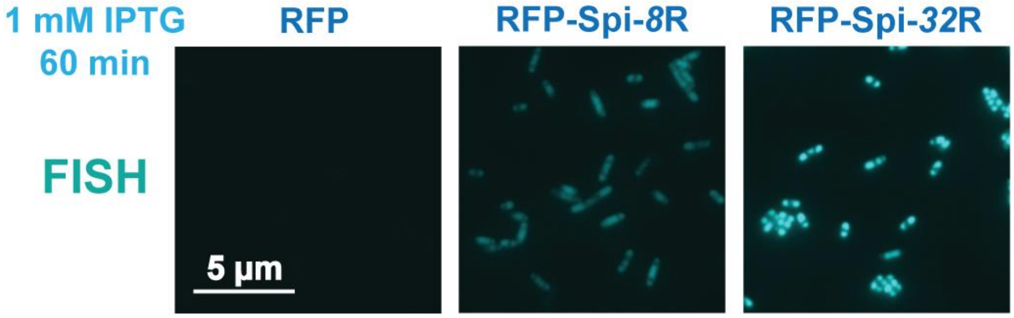

Figure S5. FISH validation of the localization of Spinach-tagged RFP mRNA, using FISH probes against the Spinach aptamer.

Figure S6

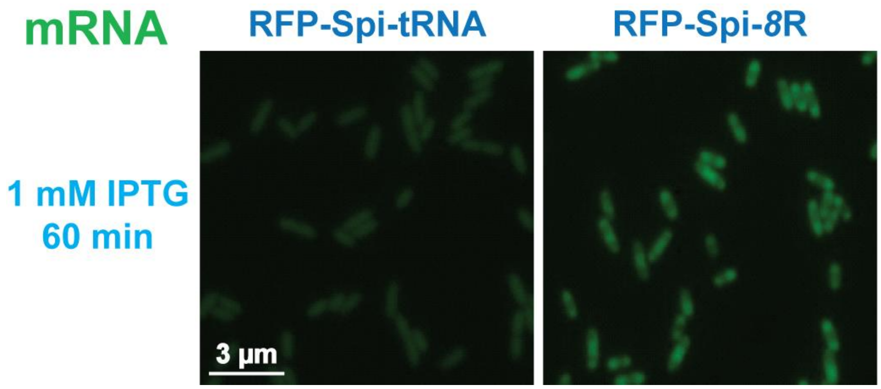

Figure S6. Representative fluorescence images of *E. coli* expressing mRNA tagged by Spi-tRNA and Spi-*8*R.

Figure S7

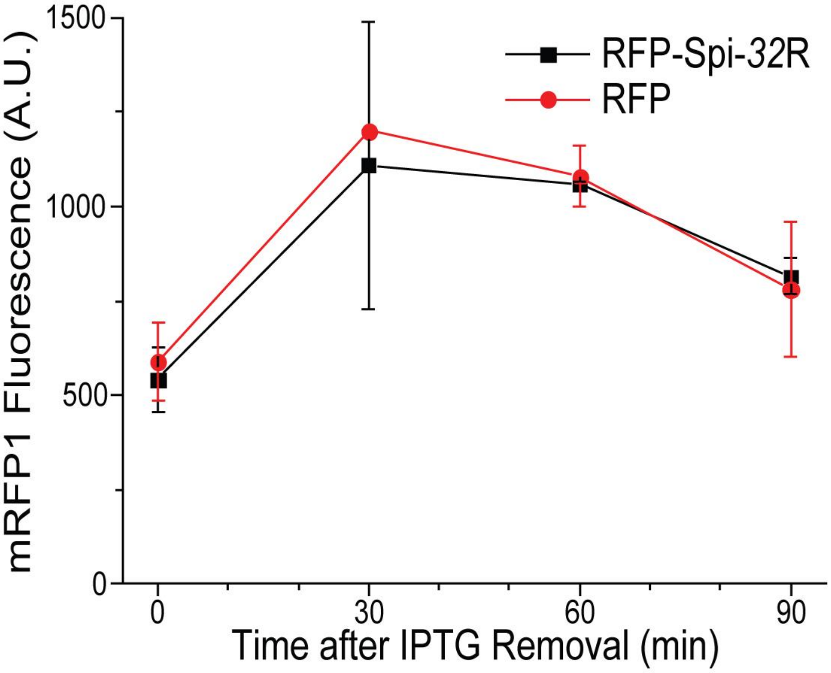

Figure S7. mRFP1 protein fluorescence level as a function of time in the decay assay, in *E. coli* expressing RFP-Spi-32R and unmodified RFP mRNA.

Figure S8

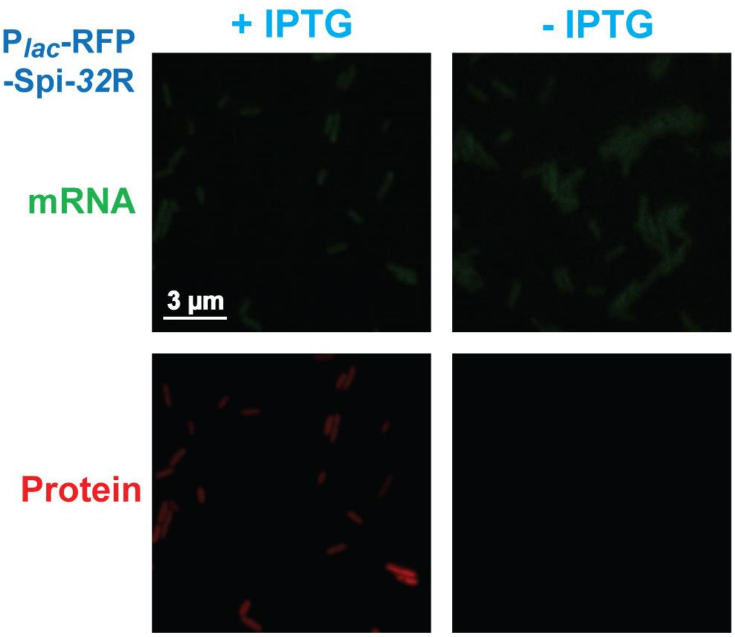

Figure S8. Representative epifluorescence images of E. coli expressing P_{lac}-RFP-Spi-32R, before and after IPTG induction.

Figure S9

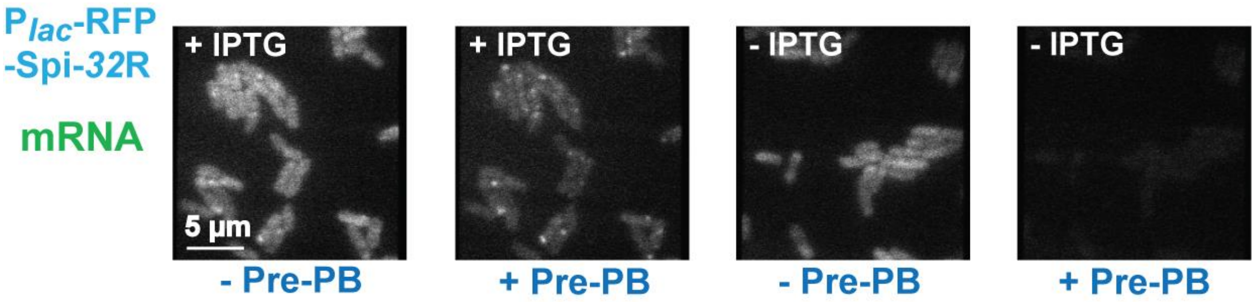

Figure S9. Fluorescence images of P_{lac}-RFP-Spi-32R cells before and after pre-photobleaching (pre-PB; CW illumination, 10 s) of autofluorescence.

Figure S10

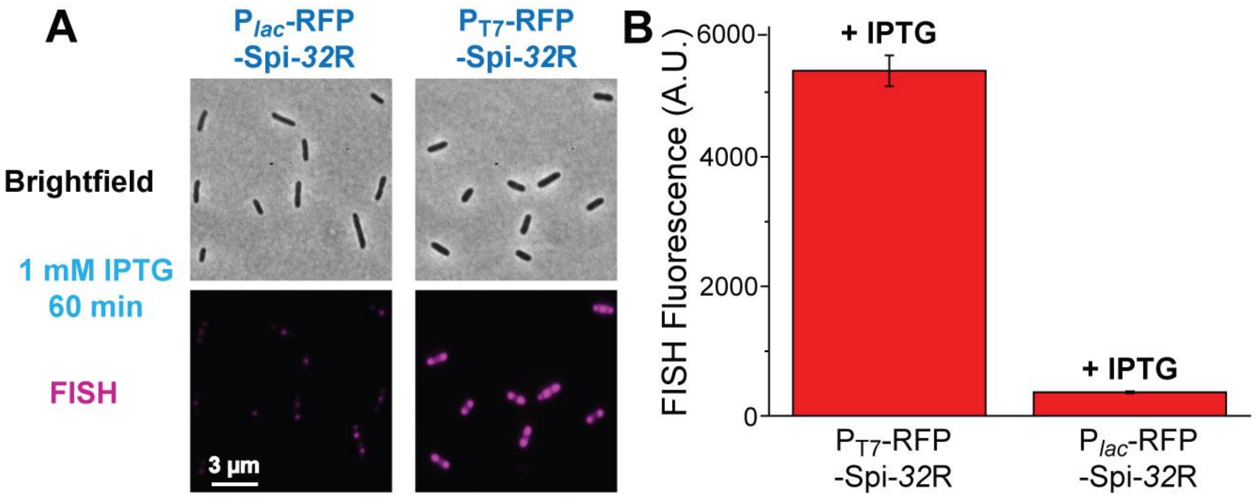

Figure S10. (A) FISH images indicating RFP-Spi-32R mRNA localization and expression level under the control of different promoters in *E. coli*. (B) Average FISH fluorescence intensity in *E. coli* expressing P_{lac}-RFP-Spi-32R and P_{T7}-RFP-Spi-32R.

Figure S11

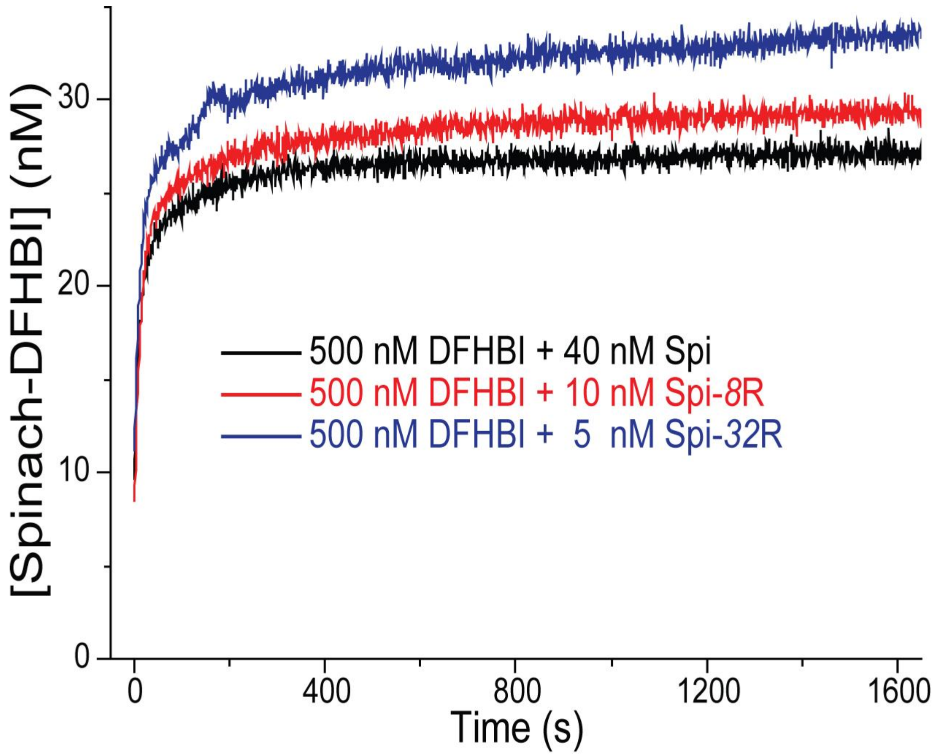

Figure S11. Representative Spinach/DFHBI binding curve for single Spinach aptamer (Spi) and tandem Spinach arrays (Spi-*8*R and Spi-32R).

Figure S12

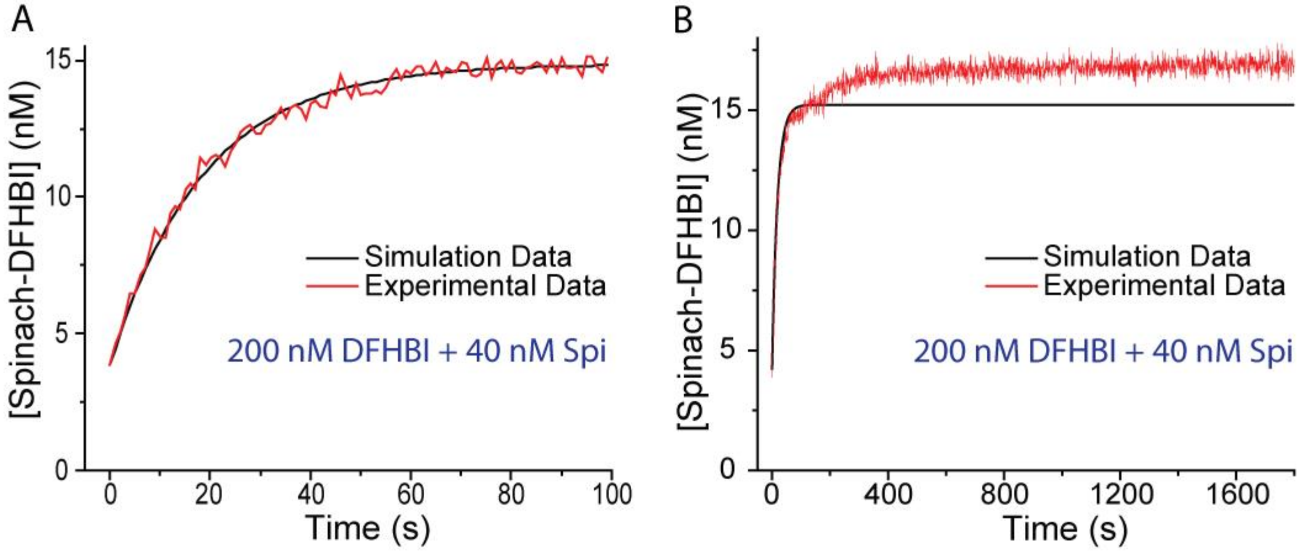

Figure S12. Representative Spinach/DFHBI binding curve (200 nM DFHBI + 40 nM Spi; red line) and the fitting curve using the estimated most probable k_{on} and k_{off} value (black line). (A) First 100 s of the binding curve and the fitting curve generated by simulating the first 100 s of the binding curve. (B) The full binding curve (1800 s) and the fitting curve using the estimated most probable k_{on} and k_{off} value generated from simulating the first 100 s of the binding curve.

Supplementary Tables

Table S1

	RNA concentration (nM)	Aptamer concentration (nM)	Fluorescence (A. U.)	Relative folding efficiency
Spi	100	100	10.6 ± 0.9	100%
Spi-8R	12.5	100	4.61 ± 0.17	43.5 ± 1.6%
Spi-32R	3.125	100	3.67 ± 0.21	34.6 ± 2.0%

Table S1. The relative folding efficiencies of Spi and Spi-nR compared to Spi-tRNA.

Table S2

	k_{on} ($M^{-1}\cdot s^{-1}$)	k_{off} (s^{-1})	K_D (nM)
5 nM Spi-32R, 200 nM DFHBI	7.9×10^4	3.1×10^{-2}	392
5 nM Spi-32R, 300 nM DFHBI	8.7×10^4	2.7×10^{-2}	310
5 nM Spi-32R, 400 nM DFHBI	6.7×10^4	2.1×10^{-2}	313
5 nM Spi-32R, 500 nM DFHBI	7.7×10^4	3.5×10^{-2}	455
5 nM Spi-32R, 600 nM DFHBI	5.7×10^4	3.5×10^{-2}	614
5 nM Spi-32R, 700 nM DFHBI	6.9×10^4	2.9×10^{-2}	420
Mean ± SD	$(7.3 \pm 1.1)\times10^4$	$(3.0 \pm 0.5)\times10^{-2}$	417 ± 112
10 nM Spi-8R, 200 nM DFHBI	10.1×10^4	2.7×10^{-2}	267
10 nM Spi-8R, 300 nM DFHBI	9.8×10^4	2.8×10^{-2}	286
10 nM Spi-8R, 400 nM DFHBI	8.9×10^4	2.9×10^{-2}	326
10 nM Spi-8R, 500 nM DFHBI	9.3×10^4	1.7×10^{-2}	183
10 nM Spi-8R, 600 nM DFHBI	8.8×10^4	1.5×10^{-2}	170
Mean ± SD	$(9.4 \pm 0.6)\times10^4$	$(2.3 \pm 0.7)\times10^{-2}$	246 ± 67
40 nM Spi, 200 nM DFHBI	9.9×10^4	3.1×10^{-2}	313
40 nM Spi, 400 nM DFHBI	7.7×10^4	2.3×10^{-2}	299
40 nM Spi, 500 nM DFHBI	7.7×10^4	2.5×10^{-2}	325
40 nM Spi, 600 nM DFHBI	7.7×10^4	2.1×10^{-2}	273
Mean ± SD	$(8.3 \pm 1.1)\times10^4$	$(2.5 \pm 0.4)\times10^{-2}$	303 ± 22

Table S2. The *kon*, *koff* and K_D of DFHBI binding onto the Spinach aptamer within Spi, Spi-8R and Spi-32R, measured under various DFHBI concentration conditions.

Sequence Information

Spi

gacgcaactgaatgaaatggtgaaggacgggtccaggtgtggctgcttcggcagtgcagcttgttgagtagagtgtgagctccgtaactagtcgcgtc

Spi-tRNA

gcccggatagctcagtcggtagagcagcggccggacgcaactgaatgaaatggtgaaggacgggtccaggtgtggctgcttcggcagtgcagcttgttgagtagagtgtgag
ctccgtaactagtcgcgtccggccgcgggtccagggttcaagtccctgttcgggcgcca

(red letters refer to tRNA sequence; green letters refer to Spinach aptamer sequence, or 24-2 sequence)

Spi-*8*R

tagacggcatggggagacgcgaccgaaatggtgaaggacgggtccagtgcttcggcactgttgagtagagtgtgagctccgtaactggtcgcgtcacgtaagatgctccggtt
agggagacgcgaccgaaatggtgaaggacgggtccagtgcttcggcactgttgagtagagtgtgagctccgtaactggtcgcgtcactgatgtaccgttgagcagggagacg
cgaccgaaatggtgaaggacgggtccagtgcttcggcactgttgagtagagtgtgagctccgtaactggtcgcgtcactcgctagagcatggtttgggagacgcgaccgaaat
ggtgaaggacgggtccagtgcttcggcactgttgagtagagtgtgagctccgtaactggtcgcgtcactggggcacgccgtctgggggagacgcgaccgaaatggtgaagga
cgggtccagtgcttcggcactgttgagtagagtgtgagctccgtaactggtcgcgtcacttactgcgaccgcaatagggagacgcgaccgaaatggtgaaggacgggtccagt
gcttcggcactgttgagtagagtgtgagctccgtaactggtcgcgtcacgcgcgcaaccgggtagagggagacgcgaccgaaatggtgaaggacgggtccagtgcttcggca
ctgttgagtagagtgtgagctccgtaactggtcgcgtcacgtaactcacggcgctatgggagacgcgaccgaaatggtgaaggacgggtccagtgcttcggcactgttgagta
gagtgtgagctccgtaactggtcgcgtc

(black letters refer to linker sequence between Spinach aptamers; green letters refer to Spinach aptamer (minimal) sequence, or 24-2-min sequence)

Spi2-*8*R

tagacggcatggggagatgtaactgaatgaaatggtgaaggacgggtccagtaggctgcttcggcagcctacttgttgagtagagtgtgagctccgtaactagttacatcacgt
aagatgctccggttagggagatgtaactgaatgaaatggtgaaggacgggtccagtaggctgcttcggcagcctacttgttgagtagagtgtgagctccgtaactagttacatc
actgatgtaccgttgagcagggagatgtaactgaatgaaatggtgaaggacgggtccagtaggctgcttcggcagcctacttgttgagtagagtgtgagctccgtaactagtta
catcactcgctagagcatggtttgggagatgtaactgaatgaaatggtgaaggacgggtccagtaggctgcttcggcagcctacttgttgagtagagtgtgagctccgtaacta
gttacatcactggggcacgccgtctgggggagatgtaactgaatgaaatggtgaaggacgggtccagtaggctgcttcggcagcctacttgttgagtagagtgtgagctccgta
actagttacatcacttactgcgaccgcaatagggagatgtaactgaatgaaatggtgaaggacgggtccagtaggctgcttcggcagcctacttgttgagtagagtgtgagctc
cgtaactagttacatcacgcgcgcaaccgggtagagggagatgtaactgaatgaaatggtgaaggacgggtccagtaggctgcttcggcagcctacttgttgagtagagtgtg
agctccgtaactagttacatcacgtaactcacggcgctatgggagatgtaactgaatgaaatggtgaaggacgggtccagtaggctgcttcggcagcctacttgttgagtagag
tgtgagctccgtaactagttacatc

(black letters refer to linker sequence between “Spinach 2” aptamers; green letters refer to “Spinach 2” aptamer sequence)

mRFP1

atggcctcctccgaggacgtcatcaaggagttcatgcgcttcaaggtgcgcatggagggctccgtgacacgagttcgagatcagagggcgagggccgcccctacgagggcac
ccagaccgccaagctgaaggtgaccaagggcggcccccctgcccttcgcctgggacatcctgtcccctcagttccagtacggctccaaggcctacgtgaagcaccccgccgac
atccccgactacttgaagctgtccttccccgagggcttcaagtgggagcgcgtgatgaacttcgaggacggcggcgtggtgaccgtgacccaggactcctccctgcaggacgg
cgagttcatctacaaggtgaagctgcgcggcaccaacttcccctccgacggccccgtaatgcagaagaagaccatgggctgggaggcctccaccgagcggatgtaccccgag
gacggcgccctgaagggcgagatcaagatgaggctgaagctgaaggacggcggccactacgacgccgaggtcaagaccacctacatggccaagaagcccgtgcagctgcc
cggcgcctacaagaccgacatcaagctggacatcacctcccacaacgaggactacaccatcgtggaacagtacgagcgcgccgagggccgccactccaccggcgcctaa

T7 promoter-*lac* operator

taatacgactcactataggggaattgtgagcggataacaattc

(red letters refer to T7 promoter sequence, and green letters refer to *lac* operator sequence)

***lac* promoter-*lac* operator**

tttacactttatgcttccggctcgtatgttgtgtggaattgtgagcggataacaatttc

(blue letters refer to *lacZYA* promoter sequence, and green letters refer to *lac* operator sequence)

RNA FISH probes

mRFP1 coding sequence

5'-tgatgacgtcctcggaggag-3'
5'-accttgaagcgcatgaactc-3'
5'-tgatctcgaactcgtgtcac-3'
5'-tactggaactgaggggacag-3'
5'-gggaaggacagcttcaagta-3'
5'-cgtcctcgaagttcatcacg-3'
5'-gcttcaccttgtagatgaac-3'
5'-atggtcttcttctgcattac-3'
5'-ttcagcctcatcttgatctc-3'
5'-atgtaggtggtcttgacctc-3'
5'-cttgatgtcggtcttgtagg-3'
5'-tcgttgtgggaggtgatgtc-3'
3'-atgtaggtggtcttgacctc-3'

Spinach aptamer

5'-aagcactggacccgtccttc-3'
5'-cacactctactcaacagtgc-3'
5'-tgacgcgaccagttacggag-3'

qPCR primers

mRFP1

Forward: 5'-tgaggctgaagctgaaggac-3'
Reverse: 5'-tgtccagcttgatgtcggtc-3'

16S rRNA

Forward: 5'-aggccttcgggttgtaaagt-3'
Reverse: 5'-attccgattaacgcttgcac-3'

Reference

1 Paige, J. S., Wu, K. Y. & Jaffrey, S. R. RNA mimics of green fluorescent protein. *Science* **333**, 642-646, doi:10.1126/science.1207339 (2011).
2 Golding, I. & Cox, E. C. RNA dynamics in live Escherichia coli cells. *Proc Natl Acad Sci U S A* **101**, 11310-11315, doi:10.1073/pnas.0404443101 (2004).
3 Golding, I., Paulsson, J., Zawilski, S. M. & Cox, E. C. Real-time kinetics of gene activity in individual bacteria. *Cell* **123**, 1025-1036, doi:10.1016/j.cell.2005.09.031 (2005).

4 Roy, R., Hohng, S. & Ha, T. A practical guide to single-molecule FRET. *Nat Methods* **5**, 507-516, doi:10.1038/nmeth.1208 (2008).
5 Schmittgen, T. D. & Livak, K. J. Analyzing real-time PCR data by the comparative C(T) method. *Nat Protoc* **3**, 1101-1108 (2008).
6 Gourse, R. L., Gaal, T., Bartlett, M. S., Appleman, J. A. & Ross, W. rRNA transcription and growth rate-dependent regulation of ribosome synthesis in Escherichia coli. *Annu Rev Microbiol* **50**, 645-677, doi:10.1146/annurev.micro.50.1.645 (1996).
7 Neidhardt, F. C., Ingraham, J. L. & Schaechter, M. *Physiology of the Bacterial cell: A Molecular Approach*. (Sinauer associates, 1990).
8 Raj, A., van den Bogaard, P., Rifkin, S. A., van Oudenaarden, A. & Tyagi, S. Imaging individual mRNA molecules using multiple singly labeled probes. *Nat Methods* **5**, 877-879, doi:10.1038/nmeth.1253 (2008).
9 So, L. H. *et al.* General properties of transcriptional time series in Escherichia coli. *Nat Genet* **43**, 554-560, doi:10.1038/ng.821 (2011).
